# Genome Sequence of a Central Chimpanzee-Associated Polyomavirus Related to BK and JC Polyomaviruses, *Pan troglodytes troglodytes* Polyomavirus 1

**DOI:** 10.1128/genomeA.00888-15

**Published:** 2015-09-03

**Authors:** Nadège F. Madinda, Martha M. Robbins, Christophe Boesch, Fabian H. Leendertz, Bernhard Ehlers, Sébastien Calvignac-Spencer

**Affiliations:** a Epidemiology of Highly Pathogenic Microorganisms, Robert Koch Institute, Berlin, Germany; b Department of Primatology, Max Planck Institute for Evolutionary Anthropology, Leipzig, Germany; c Institut de Recherches en Ecologie Tropicale, Libreville, Gabon; d FG12 “Measles, Mumps, Rubella and Viruses Affecting Immunocompromised Patients,” Robert Koch Institute, Berlin, Germany

## Abstract

We amplified and sequenced the genome of a polyomavirus infecting a central chimpanzee (*Pan troglodytes troglodytes*). This virus, which is closely related to BK and JC polyomaviruses, may help shed a new light on these human pathogens’ evolutionary history.

## GENOME ANNOUNCEMENT

As many as 13 distinct polyomaviruses (family *Polyomaviridae*) have been detected from human tissues (1), and a number of close relatives to these viruses have been identified in African great apes (2–5). We recently amplified and sequenced a ca. 200-bp VP1 fragment from a fecal sample collected from a wild central chimpanzee (*Pan troglodytes troglodytes*) in the Loango National Park, Gabon. This short sequence proved to be closely related to BK and JC polyomaviruses (BKPyV and JCPyV, respectively), which were until then associated only with PyV infecting monkeys. Here, we report the whole genome sequence of this virus, tentatively named *Pan troglodytes troglodytes* polyomavirus 1 (PtrotPyV1) (5).

We amplified and sequenced an additional ca. 500-bp VP1 fragment of this virus DNA. The consensus of the two short sequences was used to design outward-directed primers with which we amplified the rest of the genome. We selected 2 PCR products derived from separate DNA extracts and sequenced them by primer walking. Both products showed identical sequences.

The complete circular genome comprises 5,159 bp and exhibits the typical organization of polyomaviruses, with an early region encoding small T and large T antigens and a late region encoding VP1, VP2, and VP3 proteins. Like other members of the clade comprising BKPyV and JCPyV, PtrotPyV1 also encodes an agnoprotein. A BLAST search revealed this virus is most similar to—but undoubtedly distinct from—BKPyV (82% overall identity). Phylogenetic analyses suggested PtrotPyV1 as the sister taxon to a clade comprising BKPyV and all other related PyV infecting nonhuman primates (http://dx.doi.org/10.6084/m9.figshare.1456082).

As chimpanzees are well-known hunters and often target monkeys, we sought to confirm the association of PtrotPyV1 with its putative chimpanzee host (and not with a potential digested prey) and performed a molecular diet analysis (6). No trace of monkey or other vertebrate DNA was detected in this fecal DNA extract. We also used a specific PCR to screen 65 central chimpanzee and 64 western lowland gorilla (*Gorilla gorilla gorilla*) fecal samples collected in the same national park. No sample was found positive.

All in all, it seems plausible that PtrotPyV1 infects central chimpanzees and is shed, albeit rarely, in their feces. Further screening of wild chimpanzee communities should allow reinforcing of this preliminary conclusion. In addition, novel PyV belonging to this group may be detected from fecal material of other wild great apes and, together with PtrotPyV1, contribute to enlighten the evolutionary history of BKPyV and JCPyV. We expect that the identification of such viruses will notably help clarify the role of codivergence in the evolution of this PyV lineage (7).

### Nucleotide sequence accession number.

The complete genome of PtrotPyV1 was deposited in GenBank under the accession number KT184855.
